# FACTORS ASSOCIATED WITH MEANINGFUL ACTIVITIES AMONG ETHNICALLY DIVERSE OLDER ADULTS

**DOI:** 10.1093/geroni/igz038.1905

**Published:** 2019-11-08

**Authors:** Dolapo O Adeniji, Michin Hong

**Affiliations:** 1 School of Social work, Indiana Purdue University Indianapolis, Indianapolis, Indiana, United States; 2 Indiana University, Indianapolis, Illinois, United States

## Abstract

Objectives: With aging, there is an increased chance for older adults to experience negative health outcome and lose independence. Previous studies have shown the positive influence of engagement in physical, religious, recreational and social activities on healthy aging. This study aims to examine the factors contributing to the frequency of activity participation that helps older adults achieve the goal of staying healthy. Method: A sample (n=480) aged 60-90 with M=74.31, SD=7.65, and female 55.6 % from Well Elderly II were surveyed for the study. Hierarchical regression analysis was performed to examine factors associated with the frequency of activity participation. Age, ethnicity, education and gender controlling for meaning ascribed to activity were entered in model I. While the independent variables: social support, perceived mental and physical health and meaning ascribed for activity participation were in model II. Model II (R2=57.8%) significantly improved the prediction of the outcome variable greater than model I (R2=13.4%). Results: Results showed that age, education, ethnicity, perceived mental health, physical health, social support were significant. Of all variables the meaning attributed to activity participation (β =59.2%, <.001) is the most predictor of meaningful frequency of activity participation in older adults. This implies that the meaning individuals attribute to activity participation increases their frequency of activity participation. Also, age, education, and ethnicity play important role in understanding the importance of consistency in activity participation. Implications: Meaningful activity participation should be encouraged to enable older adults increase their participation in activities that promote healthy living.

